# Supplementary material for: A Convoy of Magnetic Millirobots Transports Endoscopic Instruments for Minimally‐Invasive Surgery
Source: Adv Sci (Weinh). 2024 Jul 1;11(35):2308382. doi: 10.1002/advs.202308382 (PMC12462913; doi:10.1002/advs.202308382)
Supplement: Supplementary file 1 — Supporting Information [file ADVS-11-2308382-s004.docx]

Supporting Information

A convoy of magnetic millirobots transports endoscopic instruments for minimally-invasive surgery

Moonkwang Jeong^+^, Xiangzhou Tan^+^, Felix Fischer and Tian Qiu*

M. Jeong

Cyber Valley group - Biomedical Microsystems, Institute of Physical Chemistry, University of Stuttgart, Pfaffenwaldring 55, 70569 Stuttgart, Germany

X. Tan
Department of General Surgery, Xiangya Hospital, Central South University, Changsha 410008, China

International Joint Research Center of Minimally Invasive Endoscopic Technology Equipment & Standards, Changsha 410008, China

F. Fischer
Division of Smart Technologies for Tumor Therapy, German Cancer Research Center (DKFZ) Site Dresden, Blasewitzer Str. 80, 01307 Dresden, Germany

Faculty of Engineering Sciences, University of Heidelberg, Heidelberg, Germany

T. Qiu

Division of Smart Technologies for Tumor Therapy, German Cancer Research Center (DKFZ) Site Dresden, Blasewitzer Str. 80, 01307 Dresden, Germany

Faculty of Medicine Carl Gustav Carus, Technical University Dresden, Germany

Faculty of Electrical and Computer Engineering, Technical University Dresden, Germany

+ These authors contribute equally to this work.

* Corresponding author, e-mail: tian.qiu@dkfz.de


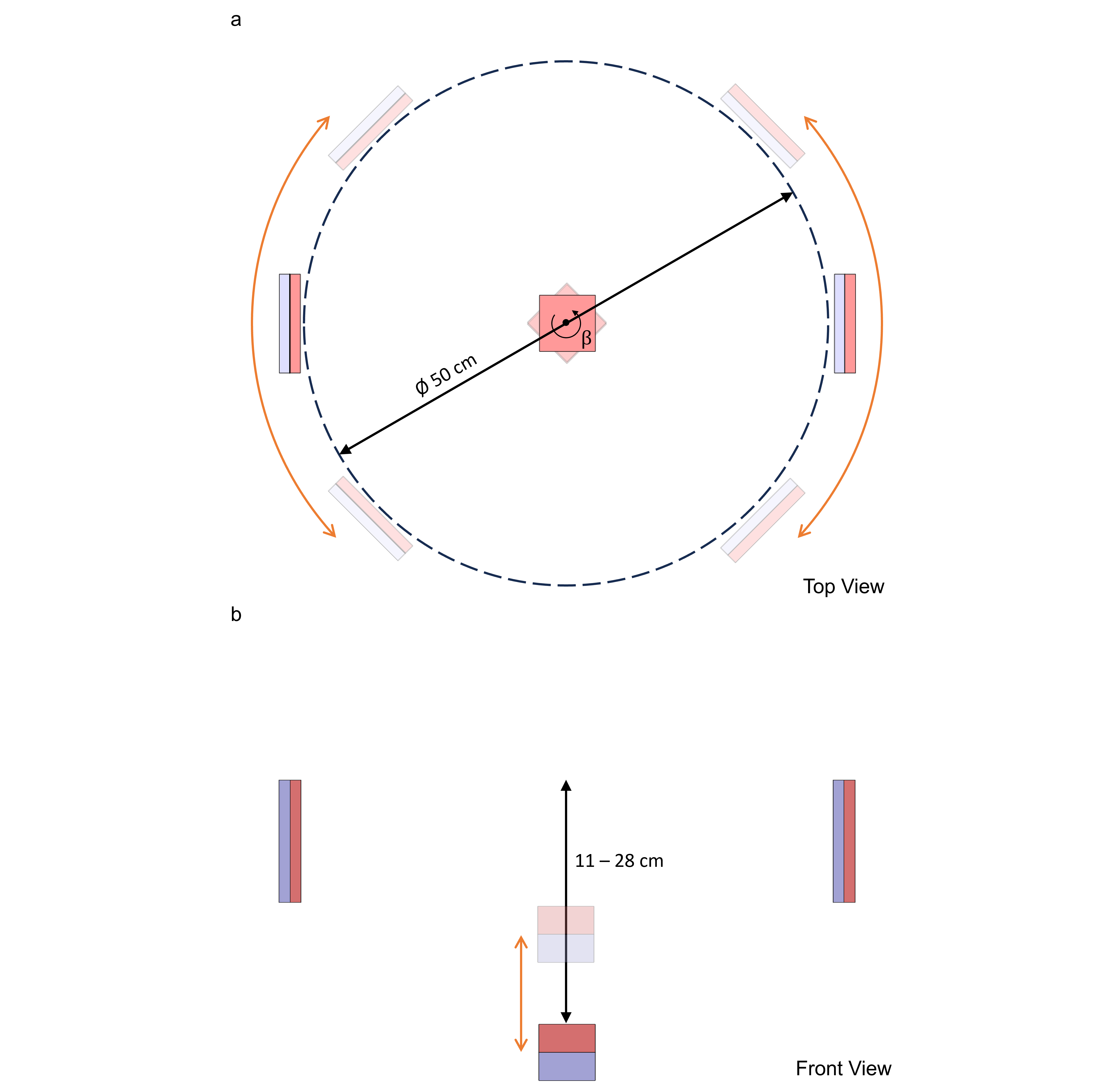


**Figure S1.** (a) Top view and (b) side view of the permanent magnetic set-up to show the accessible volume (max. Ø 50 cm × 28 cm).


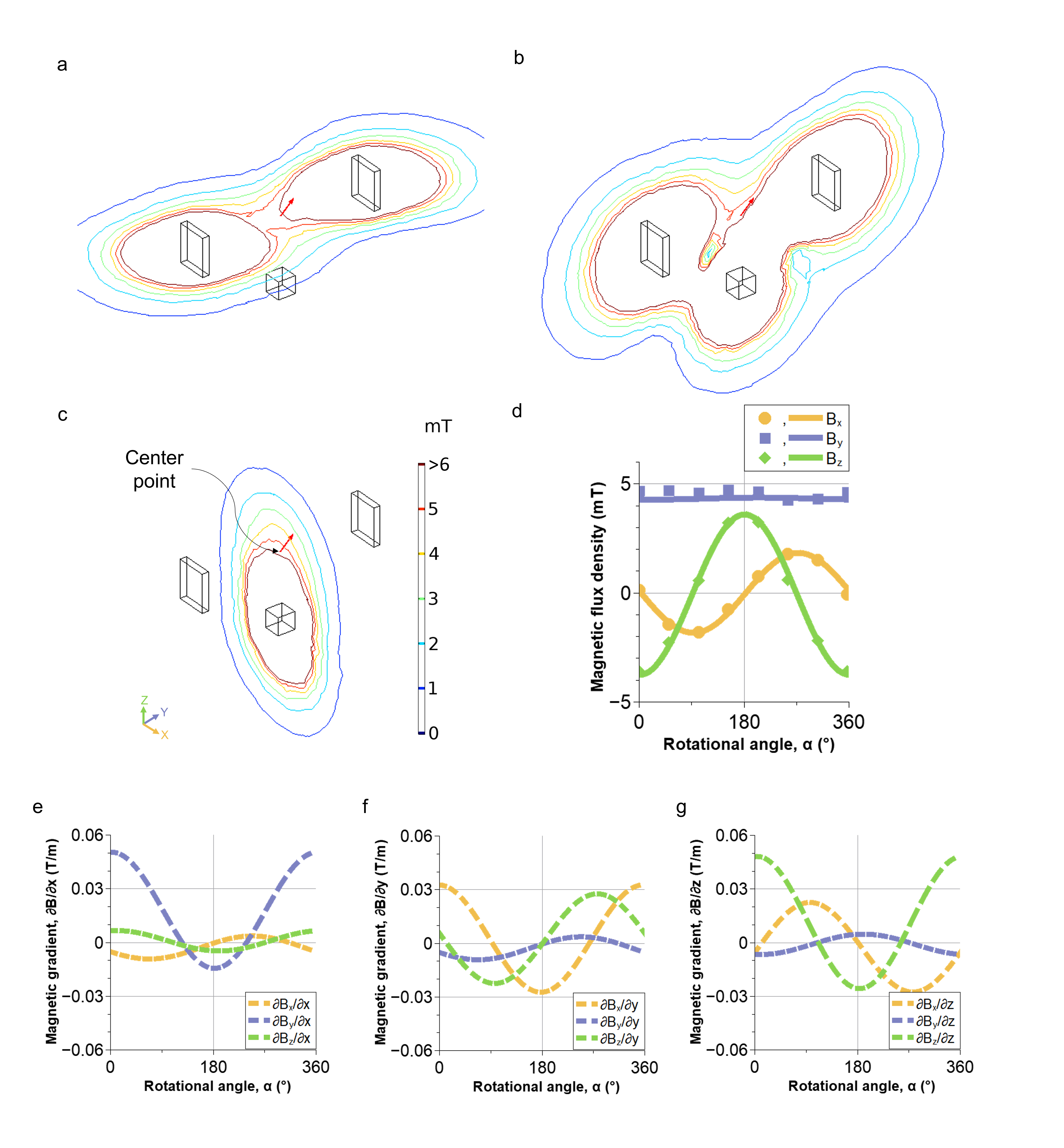


**Figure S2.** Numerical simulation results of the magnetic flux density on (a) *xy*-plane, (b) *yz*-plane, and (c) *xz*-plane when 𝛼 = 180° with the red arrow indicating the magnetic field direction at the center point. Numerical simulation results during a full cycle of (d) magnetic flux density (dots indicate experimental results and solid lines indicate the simulation results), and (e-g) magnetic gradient at the center point in *x*-, *y*- and *z*-direction, respectively.


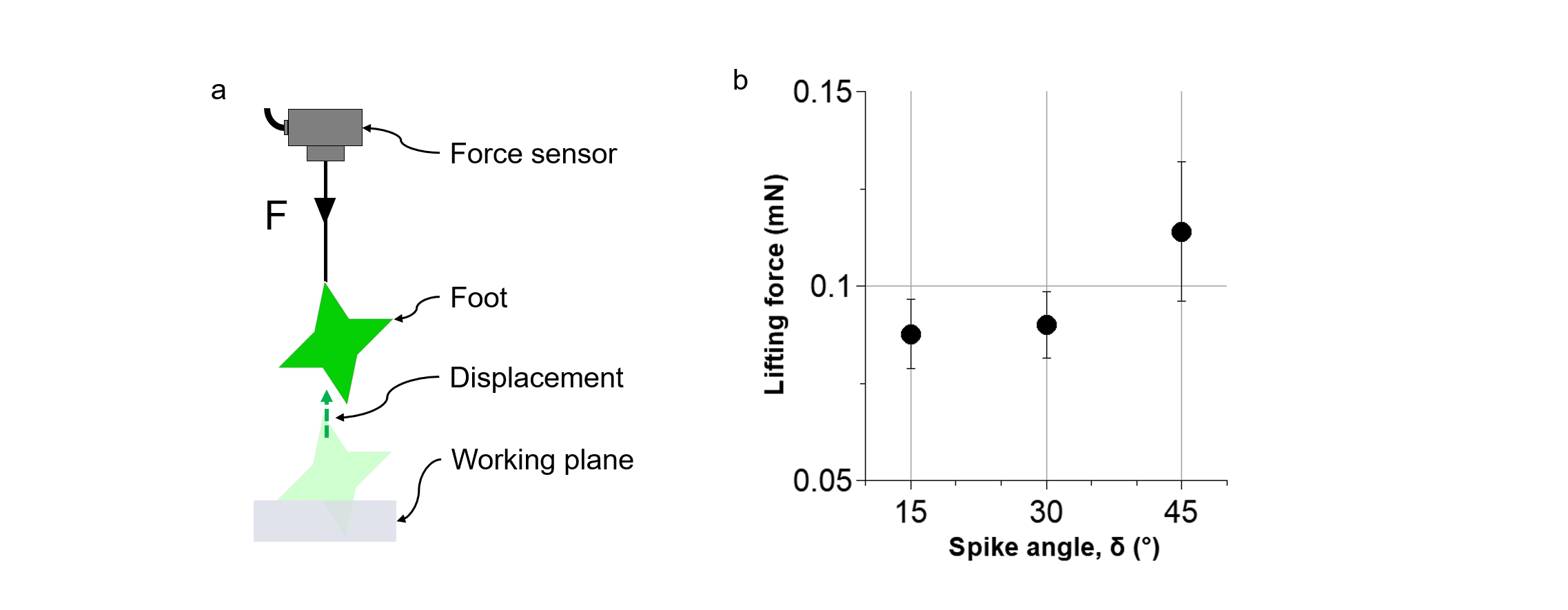


**Figure S3.** Lifting force measurement of the foot designs. (a) Schematic of the simplified lifting force measurement set-up. A foot is attached to a force sensor by a wire and lifted from a hydrogel phantom in the vertical direction. (b) Lifting force measured on the different spike angles 𝛿.


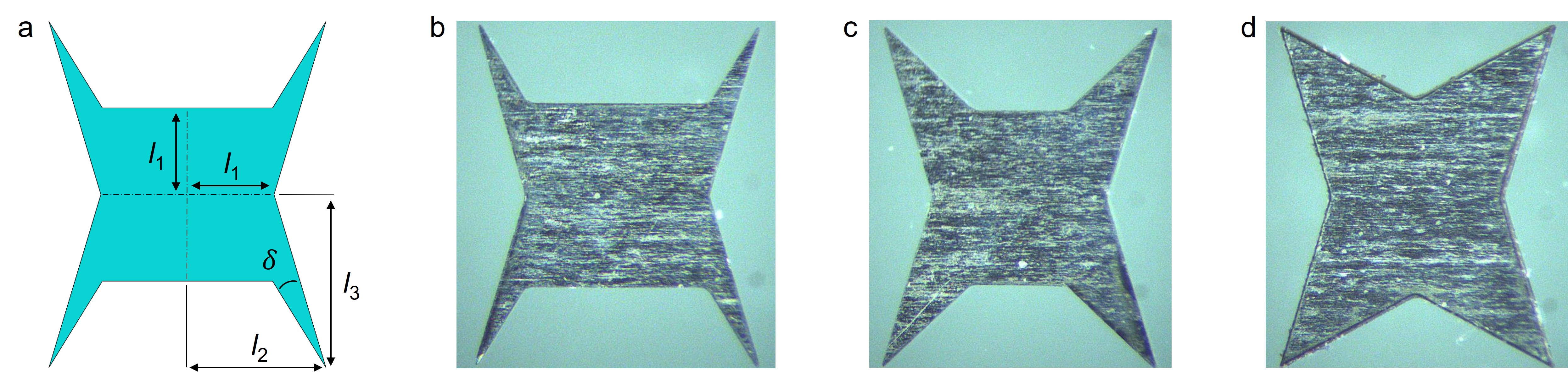


**Figure S4.** Fabrication of the feet with spikes. (a) Design of a foot (*l*_1_ = 0.5 mm, *l*_2_ = 0.8 mm and *l*_3_ = 1 mm). Microscopic images of different designs: (b) 𝛿 = 15°, (c) 𝛿 = 30° and (d) 𝛿 = 45°.


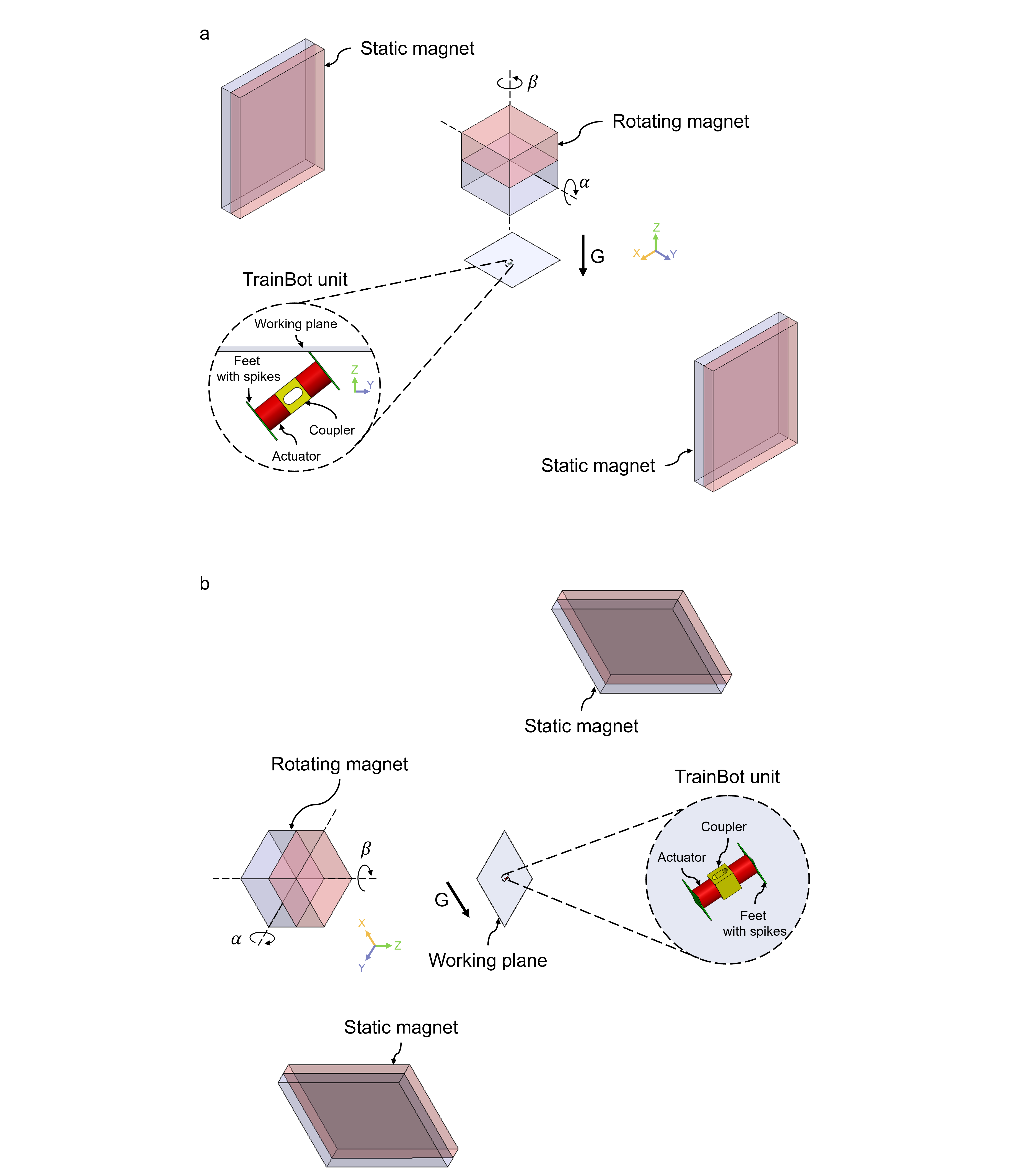


**Figure S5.** Configuration of the magnetic actuation set-up for (a) upside-down crawling and (b) vertical crawling. The black arrow with the letter “G” indicates the direction of gravity.


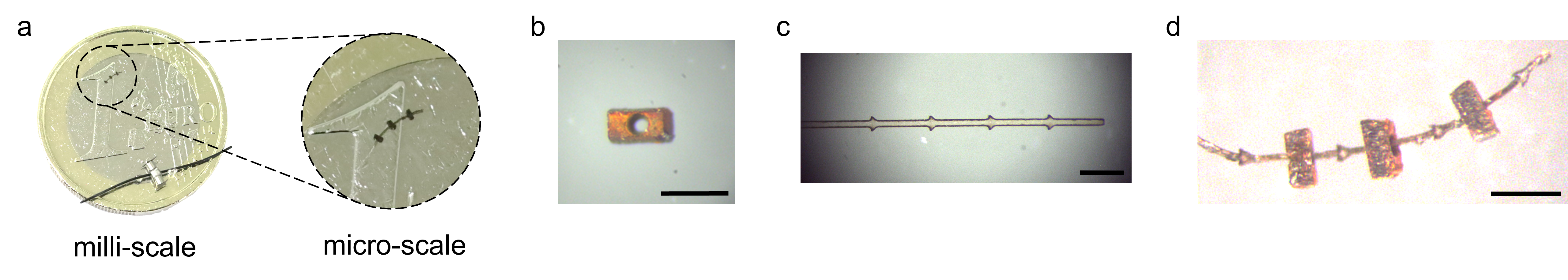


**Figure S6.** The sub-millimeter scale TrainBot. (a) Picture of TrainBots in milli- and micro-scale on a coin. A micro-scale TrainBot consists of (b) three microrobots and (c) a customized wire connected through couplers in the middle of each TrainBot unit. (d) Microscopic image of assembled sub-millimeter scale TrainBot. All scale bars are 500 μm.


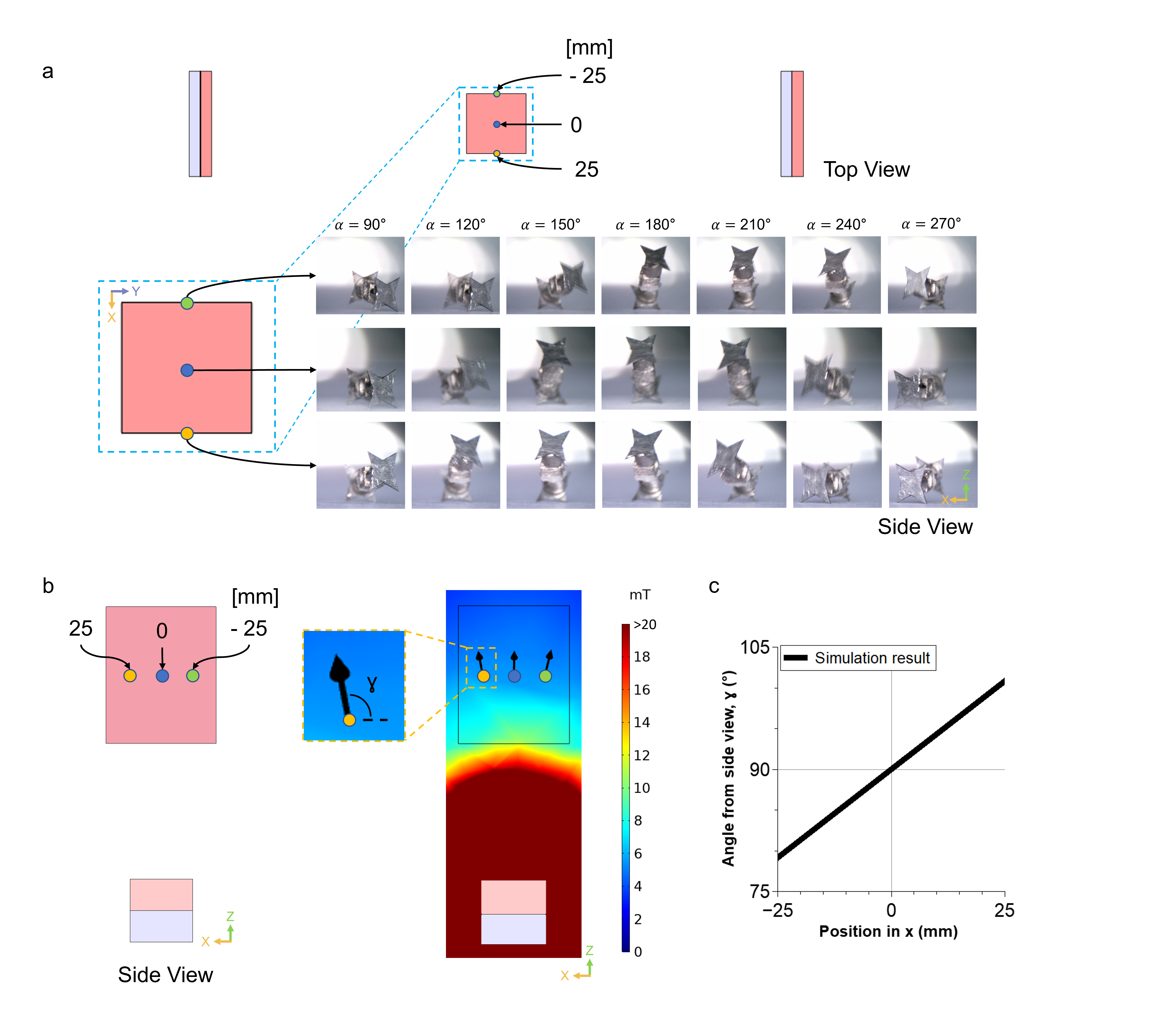


**Figure S7.** Phase lag measurement of the locomotion of a Trainbot unit. (a) Schematic top view of the magnetic actuation set-up and image sequences captured from the side view at different locations (at -25 mm, 0 mm, and 25 mm). (b-c) Simulation result of the angle *γ* at different locations.


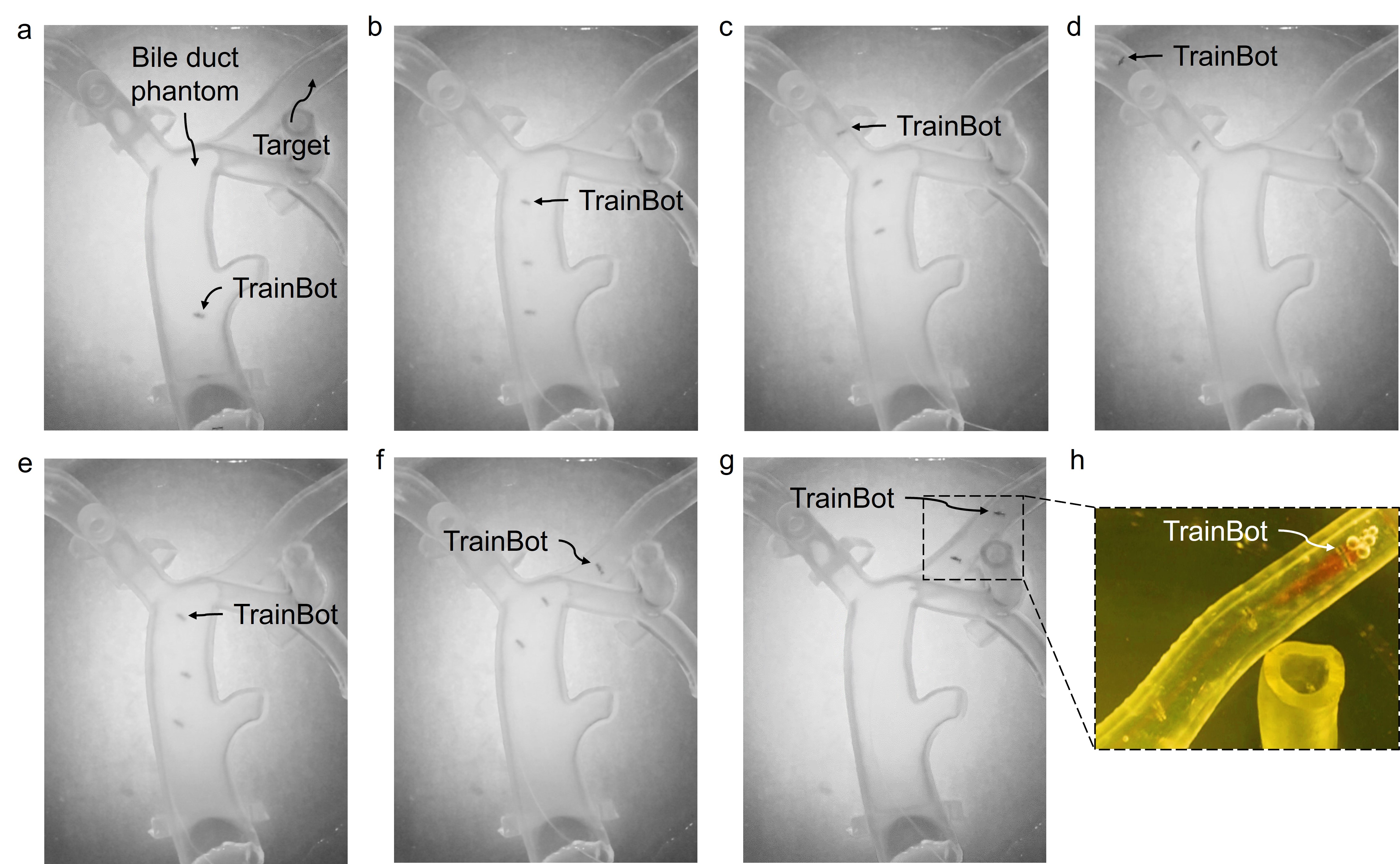


**Figure S8.** Control and targeted drug delivery experiment of the TrainBot in a human-scale bile duct phantom. TrainBot carries a catheter (OD: 0.5 mm, ID: 0.3 mm). (a) The TrainBot is inserted in the common bile duct and (b-d) actuated to reach the left bile duct. (e) The TrainBot is actuated to move back to the common bile duct and (f-g) towards the target on the right side of the bile duct for (h) drug delivery.
